# 49483 Evaluating the Role of IFNLR1 Receptor Dynamics and Plasticity in Regulating Cellular Response to Interferons

**DOI:** 10.1017/cts.2021.440

**Published:** 2021-03-30

**Authors:** Gray Evans, Christiana S. Kappler, Ray Liu, Juliana D. Carten, Cody M. Orr, Sarah Stephenson, Paula Traktman, Stephen A. Duncan, Eric G. Meissner

**Affiliations:** MUSC

## Abstract

ABSTRACT IMPACT: We hope to provide a more nuanced understanding of the type-III IFN system, thereby exploring its therapeutic potential in the realm of infectious diseases. OBJECTIVES/GOALS: The role of IFNLR1 receptor dynamics and plasticity in regulating the type-III IFN response is largely unknown. As a specific, powerful component of innate immunity, understanding how the type-III IFN system is regulated could lead to the development of novel therapeutic targets and strategies to face a multitude of viral illnesses. METHODS/STUDY POPULATION: To facilitate our investigation, we will generate doxycycline-inducible FLAG-tagged IFNLR1-expression plasmids representing all known transcriptional variants. These plasmids will allow us to: 1) Evaluate the effect of IFNLR1 surface abundance on the type-III IFN transcriptional profile and 2) Assess the extent of IFNLR1-FLAG co-localization with several notable intracellular structures using immunofluorescence, before and after stimulation with IFNL3. RESULTS/ANTICIPATED RESULTS: We have successfully generated three IFNLR1-FLAG transcriptional variants and confirmed inducible-expression and function in vitro. We are currently assessing the role of surface abundance, internalization, differential isoform expression, and trafficking. DISCUSSION/SIGNIFICANCE OF FINDINGS: By completing this study, we hope to provide a more nuanced understanding of the type-III IFN system, thereby exploring its therapeutic potential in the realm of infectious diseases.

